# Severe viral respiratory infections in the pre‐COVID era: A 5‐year experience in two pediatric intensive care units in Italy

**DOI:** 10.1111/irv.13038

**Published:** 2022-10-03

**Authors:** Maia De Luca, Carmen D'Amore, Lorenza Romani, Costanza Tripiciano, Vitangelo Clemente, Stefania Mercadante, Daniela Perrotta, Joseph Nunziata, Corrado Cecchetti, Emanuele Rossetti, Roberto Bianchi, Carlo Federico Perno, Paola Bernaschi, Cristina Russo, Laura Lancella, Massimiliano Raponi, Marta Luisa Ciofi degli Atti

**Affiliations:** ^1^ Academic Department of Pediatrics (DPUO), Infectious Disease Unit, Bambino Gesù Children's Hospital IRCCS Rome Italy; ^2^ Clinical Pathways and Epidemiology Unit, Bambino Gesù Children's Hospital IRCCS Rome Italy; ^3^ Academic Department of Pediatrics University of Rome Tor Vergata Rome Italy; ^4^ Pediatric Emergency Department, Bambino Gesù Children's Hospital IRCCS Rome Italy; ^5^ Department of Anesthesia and Critical Care, Bambino Gesù Children's Hospital IRCCS Rome Italy; ^6^ Pediatric Emergency Department Pediatric Intensive Care Unit, Bambino Gesù Children's Hospital IRCCS Rome Italy; ^7^ Pediatric Intensive Care Unit, Paediatric Emergency, Anaesthesia and Intensive Care Department, Bambino Gesù Children's Hospital IRCCS Rome Italy; ^8^ Microbiology and Diagnostic Immunology Unit, Bambino Gesù Children's Hospital IRCCS Rome Italy; ^9^ Multimodal Medicine Research Area, Bambino Gesù Children's Hospital Istituto di Ricovero e Cura a Carattere Scientifico (IRCCS) Rome Italy; ^10^ Medical Direction, Bambino Gesù Children's Hospital IRCCS Rome Italy

**Keywords:** bronchiolitis, intensive care units, pediatric, respiration, artificial, respiratory insufficiency, risk factors, viruses

## Abstract

**Background:**

Viral respiratory infections are one of the main causes of hospitalization in children. Even if mortality rate is low, 2% to 3% of the hospitalized children need mechanical ventilation. Risk factors for admission to the pediatric intensive care unit (PICU) are well known, while few studies have described risk factors for invasive ventilator support and prolonged hospitalization.

**Methods:**

A retrospective study including all patients aged between 2 and 18 months with a confirmed viral respiratory infection, requiring admission to PICU from September to March between 2015 and 2019, was conducted at Bambino Gesù Children's Hospital in Rome, Italy.

**Results:**

One hundred ninety patients were enrolled, with a median age of 2.7 months; 32.1% had at least one comorbidity, mainly prematurity. The most frequent isolated viruses were RSV‐B, rhinovirus, and RSV‐A; 38.4% needed mechanical ventilation. This subgroup of patients had lower median birth weight compared with patients not requiring mechanical ventilation (2800 g vs. 3180 g, *p* = 0.02); moreover, comorbidities were present in 43.8% of intubated patients and in 24.8% of patients treated with non‐invasive ventilation (*p* = 0.006). Viral coinfection did not result to be a risk factor for mechanical support, while virus–bacteria coinfection was significantly associated with mechanical ventilation (*p* < 0.001). Similar risk factors were identified for prolonged hospitalization.

**Conclusions:**

Early identification of patients who could have a sudden respiratory deterioration and need of mechanical ventilation is crucial to reduce complications due to orotracheal intubation and prolonged hospitalization in PICU. Further studies are needed to define high‐risk group of patients and to design targeted interventions.

## 1 BACKGROUND

Viral respiratory infections (VRIs) are one of the main causes of hospitalization in children.^1^ Respiratory syncytial virus (RSV) is the most common identified pathogen in VRIs. Other common etiologies include rhinovirus, enterovirus, influenza virus, metapneumovirus, and parainfluenza virus.^2^, ^3^, ^4^, ^5^ From 2020, also SARS‐COV2 has to be considered among the viruses responsible for respiratory infections in children. Coinfection with multiple viruses occurs in 20% to 40% of cases of severe pediatric VRIs.^4^, ^6^, ^7^

Infants and toddlers are more frequently affected, with a peak incidence below 6 months of age.^8^ Pediatric VRIs have a variable course, usually mild and self‐limited in immunocompetent patients; however, symptoms like respiratory distress, apnoea, hypoxemia, and dehydration can occur, requiring hospitalization in 2% to 3% of cases.^8^, ^9^

Among the hospitalized patients, 2% to 3% needs mechanical ventilation.^9^, ^10^ However, mortality in hospitalized RSV‐infected children in developed countries is less than 0.1%; this rate increases in patients affected by underlying chronic conditions.^8^, ^11^

Factors described to be associated with severe VRIs and ICU admission are age <6 weeks, low birth weight, prematurity (<37 week of gestation age), chronic lung disease, congenital heart disease, immunodeficiency, genetic/chromosomal abnormalities, cerebral palsy, and other neurological comorbidities.^12^ To date, few studies have considered risk factors for orotracheal intubation and mechanical ventilation and for prolonged hospitalization.

The current study aims at describing viral etiologies, demographic features, and clinical course of pediatric patients affected by severe VRIs requiring PICU admission in an Italian tertiary care pediatric hospital during the period 2015–2019. The secondary aim focused on the identification of predictors of invasive ventilation support, prolonged ICU stay, and prolonged overall hospitalization in order to improve knowledge and the management skills of this subset of patients.

## 2 MATERIALS AND METHODS

### 2.1 Study setting

This study was conducted at Bambino Gesù Children's Hospital (Ospedale Pediatrico Bambino Gesù, hereafter OPBG), a 607‐bed tertiary care academic hospital in the Lazio Region, Italy. OPBG had one NICU and four PICUs; data were collected from two of the four PICUs with about 612 annual admissions on average over the study period; the average length of ICUs hospitalization was equal to 7.4 days.

### 2.2 Study design and population

We conducted a retrospective descriptive study including all patients aged between 2 and 18 months with a confirmed viral infection, identified on a respiratory sample (i.e., naso‐pharyngeal swab, tracheal swab, and/or broncho‐alveolar lavage), requiring admission to PICUs from September to March between 2015 and 2019.

Patients requiring respiratory support for underlying diseases not related to the viral infection or those receiving antiviral therapy and/or intravenous immunoglobulin therapy (IVIG) within 48 h before symptoms onset were excluded.

### 2.3 Data collection

Data were collected by physicians from patients' medical charts. Information collected for each patient included age at admission, gender, gestational age, birth weight, current weight, flu vaccination, previous VRIs, comorbidities, onset of respiratory symptoms, length of hospitalization, length of ICU hospitalization, administration of low flow oxygen or high‐flow oxygen through nasal cannula (HFNC) before ICU admission, type of respiratory support received in ICU, and antiviral and antibiotic therapies. Microbiological results were reviewed for all the study population.

We defined patients experienced a “clinical cure” if both of the following criteria were met: (1) no signs or significantly reduced signs of respiratory distress and (2) no need of ventilator support or significantly reduced need of ventilation support after ICU discharge.

Death, ICU readmission within 48 h after discharge, or need for tracheostomy were considered as “clinical failure.”

All the collected data were uploaded on Research Electronic Data Capture (REDCap) database which is a secure web application for building and managing online surveys and databases, available at no charge to not‐for‐profit institutions.^13^ All the data were analyzed anonymously.

### 2.4 Statistical analysis

Patients were described according to demographic and clinical factors. Collected data were presented as count and proportions (categorical data) or median and interquartile range (IQR, continuous data). Categorical data were compared using Chi‐squared test or the Fisher's exact test, as appropriate. Continuous data were compared through the Wilcoxon rank‐sum test.

A univariate analysis was conducted to identify risk factors related to need of invasive mechanical support, the overall duration of hospitalization, and the length of ICU hospitalization. Duration of mechanical ventilation, length of ICU admission, and length of the overall hospitalization were categorized on the median values. Sex, age, weight at birth, presence of at least one comorbidity, type of comorbidity virus coinfection, and bacterial isolation were tested as risk factors for duration of mechanical ventilation, length of PICU admission, and overall length of hospitalization.

All statistical analyses were conducted using STATA 13 (Stata Corporation, College Station, Texas, USA).

## 3 RESULTS

During the study period, 190 patients required PICUs admission for VRIs. Patients were more commonly female (n = 98, 51.6%), with a median age of 2.7 months (IQR: 1.5–7.9) and a median weight of 5000 g (IQR: 4040–7000) at the time of hospital admission (Table 1). Median birth weight was 3080 g (IQR: 2600–3470). Overall, children <1 year of age accounted for 86.8% (n = 165) of the whole cohort.

**TABLE 1:** Demographic and clinical factors of patients affected by VRI admitted to PICU from September to March between 2015 and 2019

Number of patients	190
Age in months, median (range interquartile)	2.7 (1.5–7.9)
Male gender, n (%)	92 (48.4)
Weight at admission, median (range interquartile)	5000 g (4040–7000)
Birth weight, median (range interquartile)	3080 g (2600–3470)
Comorbidities, n (%):	61 (32.1)
Neuromuscular disease	6 (9.8)
Cardiovascular disease	19 (31.1)
Respiratory disease	15 (24.6)
Kidney disease	3 (4.9)
Oncohematologic disease	2 (3.3)
Immunodeficiency	1 (1.6)
Metabolic disease	1 (1.6)
Perinatal disease	20 (32.8)
Musculoskeletal disease	0 (0.0)
Endocrine disease	1 (1.6)
Other	27 (44.3)
Previous respiratory infections, n (%)	31 (16.3)
Median duration of symptoms before hospitalization, median (range interquartile)	3 (2–5)
PICU admission from Emergency Department, n (%)	85 (44.7)
PICU admission from pediatric ward, n (%)	81 (42.6)
PICU admission from another hospital, n (%)	24 (12.6)
Length of PICU hospitalization, median (range interquartile)	8 (5–15)
Length of overall hospitalization, median (range interquartile)	14 (10–26)

Sixty‐one patients (32.1%) had at least one comorbidity, and 34 of them (55.7%) had more than one comorbidity. The most frequently comorbidities were perinatal diseases (n = 20; 32.8%), cardiovascular diseases (n = 19; 31.1%), and respiratory diseases (n = 15; 24.6%).

Thirty‐one (16.3%) had already experienced at least another previous episode of respiratory infection. None patient had received influenza vaccination. The median duration of symptoms before hospital admission was 3 days (IQR: 2–5 days).

Most patients were admitted to the PICU directly from the Emergency Department (n = 85; 44.7%), whereas 42.6% of cases (n = 81) were transferred to the PICU from a pediatric ward and 12.6% (n = 24) were referred from another hospital. Median length of hospitalization in PICU was 8 days (IQR: 5–15), whereas the overall median length of hospitalization was 14 days (IQR: 10–26) (Table 1).

### 3.1 Microbiological data analysis

**FIGURE 1:**
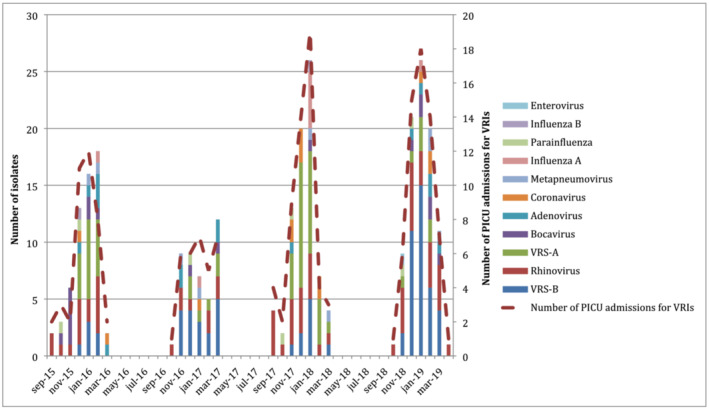
Monthly distribution of viral isolates and number of PICU admissions for VRIs

In the whole cohort, 269 detections of viral nucleic acid by RT‐PCR resulted in the respiratory tract of our patients (on average 1.4 isolates per patient). Overall, the most frequent isolated virus was RSV‐B (n = 71, 26.4%), followed by Rhinovirus (n = 66, 24.5%), RSV‐A (n = 58, 21.6%), bocavirus (n = 18, 6.7%), adenovirus (n = 16, 5.9%), coronavirus (n = 13, 4.8%), metapneumovirus (n = 9, 3.3%), influenza A (n = 8, 3.0%), parainfluenza (n = 7, 2.6%), influenza B (n = 2, 0.7%), and enterovirus (n = 1, 0.4%). Figure 1 shows the number of admissions and viral detections per month in the different autumn/winter seasons. Overall, January was the month with the higher number of PICU admission for VRIs, accounting for 34.3% of hospitalizations in 2016, 39.6% in 2018, and 43.9% in 2019. Rhinovirus was the most prevalent virus during September and October each year, while RSV was predominant since November to March. The prevalence of RSV‐A over RSV‐B changed every year but was stable during the whole season.

Of the 190 patients, 34.7% of patients had a respiratory coinfection with two or more viruses; in particular, two viruses were identified in 58 patients (30.5%), three viruses in six patients (3.2%), and four viruses in two patients (1%). The most common viral coinfections were RSV‐A or B plus rhinovirus (n = 12; 18.2%) and RSV‐A plus coronavirus (n = 7; 10.6%).

A bacterial coinfection was identified in the respiratory tract of 15.3% of patients (n = 29); *Haemophilus influenzae* was the main coinfecting pathogen (n = 12, 41.4%), followed by *Klebsiella pneumoniae* (n = 4, 13.8%) and *Staphylococcus aureus* (n = 3, 10.3%).

### 3.2 Ventilatory support

Thirty‐two patients (16.8%) received oxygen therapy in the pediatric ward before they were transferred to PICU; the median duration of oxygen therapy was equal to 12 h (IQR 6–24).

In PICU, Helmet CPAP was used in 82.6% (n = 157) of patients with a mean duration of 72 h (IQR: 36–120); among them, 54 patients (34.4%) needed an upgrade to mechanical ventilation due to worsening of the respiratory distress and ventilator parameters. Conversely, 10% (n = 19) of patients were directly intubated at the time of admission in PICU. Median duration of the mechanical ventilation in the cohort of patients that were intubated was 216 h (IQR range: 144–372). The remaining 14 patients received oxygen therapy through high‐flow nasal cannula (HFNC).

### 3.3 Antimicrobials

Overall, 264 antibiotic prescriptions were issued to 172 patients (90.5%) with a mean of 1.5 antibiotic molecules per patient; 75 patients (43.6%; 75/172) received more than one antibiotic molecules. Clarithromycin was the most prescribed antibiotic (45.8%, 121/264), followed by ceftriaxone (17%, 45/264) and amoxicillin/clavulanic acid (13.3%, 35/264). Antibiotics were equally prescribed in the group of patients that needed mechanical ventilation and in the group of patients treated with non‐invasive ventilation (91.8% vs. 89.7%, respectively; *p* = 0.6). However, intubated patients received more frequently a combined antibiotic treatment (*p* < 0.001).

Antivirals were administered in 17.8% of patients (n = 34); seven (20.6%) patients received oseltamivir for influenza infection, and 27 (79.4%) patients received ribavirin for RSV infection with a median length of therapy of 8 days (IQR: 4–15). Out of these 27 patients, 19 (70.4%) received ribavirin in association with IVIG (400 mg/kg for 3–5 days, based on patient conditions).

### 3.4 Patient outcome

The majority of our study population (n = 185; 97.4%) had a complete clinical cure. Five patients (2.6%) experienced “clinical failure”: two patients (1.0%) died, one was affected by CHD, while the other had no known comorbidities. Moreover, two patients (1.0%) needed a tracheostomy for prolonged mechanical ventilation and 1 (0.5%) needed a readmission to ICU. All the patients with a “clinical failure” had received mechanical ventilation (*p* = 0.004).

### 3.5 Risk factors for mechanical ventilation

As shown in Table 2, patients with low birth weight and with at least one comorbidity required more frequently mechanical ventilation. In particular, the median birth weight in patients requiring mechanical ventilation was 2800 g (IQR: 2470–3350) compared with 3180 g (IQR: 2710–3500) of patients not requiring mechanical ventilation (*p* = 0.02). Comorbidities were present in 43.8% (n = 32) of intubated patients and in 24.8% (n = 29) of patients treated with non‐invasive ventilation (*p* = 0.006). No differences in the type of comorbidity were found between the two groups, as well as in the viral distribution. Viral coinfection did not result to be a risk factor for mechanical support. Conversely, virus–bacteria coinfection was significantly associated with mechanical ventilation (*p* < 0.001).

**TABLE 2:** Demographic, clinical, and microbiological differences between patients who required mechanical ventilation and patients receiving non‐invasive ventilation

Variables	Patients receiving mechanical ventilation N = 73 (38.4%)	Patients receiving non‐invasive ventilation N = 117 (61.6%)	*p* value
Age in months, median (range interquartile)	2.7 (1.5–7.9)	2.5 (1.3–7.1)	0.4
Male gender, n (%)	34 (46.6)	58 (49.6)	0.7
Weight at admission, median in grams (range)	4750 (4000–6200)	5150 (4200–7500)	0.09
Birth weight, median (range)	2800 (2470–3350)	3180 (2710–3500)	0.02
Previous respiratory infections	16 (21.9)	15 (12.8)	0.09
Comorbidities	32 (43.8)	29 (24.8)	0.006
Neuromuscolar disease	5 (15.6)	1 (3.4)	0.1
Cardiovascular disease	7 (21.9)	12 (41.4)	0.1
Respiratory disease	8 (25.0)	7 (24.1)	0.9
Kidney disease	1 (3.1)	2 (6.9)	0.5
Oncohematologic disease	1 (3.1)	1 (3.4)	0.9
Immunodeficiency	0 (0.0)	1 (3.4)	0.3
Metabolic disease	1 (3.1)	0 (0.0)	0.3
Perinatal disease	12 (37.5)	8 (27.6)	0.4
Musculoskeletal disease	0 (0.0)	0 (0.0)	‐
Endocrine disease	1 (3.1)	0 (0.0)	0.3
Other	8 (25.0)	6 (20.7)	0.7
Virus type			
Adenovirus	3 (4.1)	4 (3.4)	
Bocavirus	2 (2.7)	4 (3.4)	
Coronavirus	3 (4.1)	2 (1.7)	
Influenza A	4 (5.5)	2 (1.7)	
Influenza B	0 (0.0)	1 (0.8)	0.6
Metapneumovirus	1 (1.4)	6 (5.1)	
Parainfluenza	2 (2.7)	2 (1.7)	
Rhinovirus	15 (20.5)	27 (23.1)	
RSV‐A	21 (8.8)	26 (22.2)	
RSV‐B	22 (30.1)	46 (36.7)	
Number of virus isolated			
1	43 (58.9)	81 (69.2)	
2	26 (35.6)	32 (27.3)	0.3
≥3	4 (5.5)	4 (3.4)	
Virus coinfection	30 (41.1)	39 (33.3)	0.3
Virus–bacteria coinfection	23 (31.5)	6 (5.1)	<0.001
Length of ICU hospitalization	16 (11–24)	6 (4–8)	<0.001
Length of hospitalization	27 (19–47)	11 (8–15)	<0.001

Ventilated patients had a longer length of both overall hospitalization (median: 27 days; IQR: 19–47) and PICU admission (median: 16 days; IQR: 11–24 days) compared with patients receiving non‐invasive ventilation (*p* < 0.001).

Only bacterial isolation resulted to be risk factor for prolonged mechanical ventilation: patients with bacterial isolation more frequently needed mechanical ventilation for more than 216 h (n = 14, 43.7%) with respect to those with shorter need of mechanical ventilation (n = 9, 21.9%; p = 0.04).

### 3.6 Risk factors for prolonged PICU stay and prolonged overall hospitalization

Patients with longer hospitalization had a lower median weight compared with those with shorter length of hospitalization (4735 g vs. 5400 g, *p* = 0.007). Forty patients (40.0%) experiencing longer hospitalization had at least one comorbidity with respect to those with shorter hospitalization (n = 21; 23.3%; *p* = 0.01), with a higher frequency of patients with congenital disorders (21.0% versus 6.7%, *p* = 0.005). Need for mechanical ventilation, virus coinfection, and bacterial isolation emerged to be risk factors for longer length of hospitalization (*p* ≤ 0.03).

Age less than 3 months, presence of congenital disorders, bacterial isolation, and needs for mechanical ventilation resulted to be risk factors for longer PICU hospitalization.

## 4 DISCUSSION

This study describes a cohort of 190 infants admitted to the PICU with a diagnosis of severe VRI over a 5‐year period in a tertiary care pediatric hospital in Rome, Italy.

The mean age of the patients was 2.7 months, in accordance with previous studies in the literature. In 2018, Ghazaly and Nadel described a cohort of 274 patients affected by bronchiolitis admitted to a PICU in London with a median age of 60 days (IQR 28–150 days).^13^ Moreover, in 2017, a retrospective review describing a wide cohort of patients affected by bronchiolitis in seven Australian and New Zealand hospitals showed a significant difference of the chronological age in patients requiring PICU admission compared with patients managed in the ward^14^; chronological age was, in fact, the single most important predictor of the likelihood of severe bronchiolitis.^1^

In our cohort, the median birth weight was 3080 g (range 2600–3470 g), with a significant difference (*p* = 0.02) between patients who required mechanical ventilation and patients who did not. In line with our findings, Papoff *et al* in 2011 found that infants with severe bronchiolitis had a median birth weight of 2.8 ± 0.4 kg, significantly lower than infants with mild‐to‐moderate forms not requiring ventilator support.^15^ As hypothesized by Barker *et al*, the adverse environment in utero, which affects the weight gain of the fetus, plays a role also in the reduced growth of the airways, predisposing to bronchiolar obstruction during viral infections.^16^

Among the 61 patients affected by comorbidities, 32.8% of all patients were born prematurely and 31.1% had a congenital heart disease (CHD). Even if patients with at least a comorbidity had a significantly higher need of mechanical respiratory support, neither prematurity nor CHD were statistically associated with need for mechanical ventilation, probably due to the small size of the sample. These findings confirms data from previous studies; Mecklin *et al* in 2017 showed higher risk for respiratory support in born preterm patients at less than 37 weeks and patients affected by CHD in a cohort of 105 infants with VRIs.^17^

Overall, RSV was the most frequently detected pathogen, identified in the respiratory tract of 48% of our patients. According to the literature, most children have been infected with RSV at least once by 2 years of age and present a self‐limited course of disease; however, approximately 2% to 3% of infants younger than 12 months are hospitalized with RSV infection each year in the United States.^1^ Noteworthy, in our cohort, no differences in viral distribution were noted between patients who needed mechanical ventilation and patients who did not.

Interestingly, the peak period for PICU admission due to VRI occurred between December and January in each season; this reflects the frequency of RSV, which generally peaks in Europe in the early winter season.^18^

Detection of viral coinfections occurred in 34.7% of cases in our cohort; the identification of multiple viruses in the respiratory tract did not result to be a risk factor for orotracheal intubation but seemed to be related to a longer hospitalization. Rates of viral coinfection reported in other studies range from 6% to more than 30%, but the correlation with greater disease severity and longer length of ICU staying is unclear to date. Coleman *et al* observed a similar duration of respiratory support and PICU hospitalization in patients infected with a single virus compared with patients infected with more than one virus.^19^ These data are consistent with the results published by Ghazaly and Nadel that did not find out clinical or radiologic differences between single and multiple VRIs in a cohort of 422 children.^13^ Other studies show, however, conflicting data; in particular, recently, the multivariable regression analysis of a study on 477 infants admitted to the PICU with one or multiple organisms showed an association between coinfections and higher odds of longer PICU stay, prolonged mechanical ventilation, central line requirement, and bacterial coinfection.^20^

In our study, bacterial coinfections were identified in 15.3% of children from cultural exams of the tracheal aspirates (TA) and broncho‐alveolar lavages (BAL); we demonstrated that viral–bacterial coinfection is a risk factor for mechanical ventilation (*p* < 0.001). This prevalence was lower than previously shown in other studies that reported bacterial coinfections in 26% to 45% of children with bronchiolitis admitted to PICU.^21^, ^22^ In our cohort, the rate of bacterial coinfection could be underestimate because only mechanically ventilated patients or patients with tracheostomy were tested for a bacterial coinfection. We do not routinely carry out throat cultures in infants without orotracheal intubation or tracheostomy. Consistently with other studies, in our setting, *H. influenzae* was the most frequent isolated bacterium.^23^

Despite the low rate of bacterial coinfection, antibiotics were prescribed in 90.5% of our patients, with 43.6% of patients receiving more than one antibiotic. Clarithromycin was the most common used antibiotic, followed by ceftriaxone and amoxicillin/clavulanate. The overprescription of antibiotics is probably due to the severity of the patients and to the difficulty to distinguish between a viral or bacterial respiratory infection before microbiological results are available. Bacterial pneumonia is usually diagnosed by a combination of clinical signs and symptoms, laboratory markers, and chest radiography; 53.7% of patients in our cohort had elevated values of inflammatory markers at admission, and in 59.7% of cases, chest X‐ray showed parenchymal opacities, thus making it difficult to exclude a bacterial etiology. However, the misuse and abuse of antibiotics is a challenging problem in Italy and all over the world thus antibiotic stewardship programs should aim to identify feasible targets to monitor and modify the prescription patterns in these settings.^24^

Antivirals were prescribed in the 17.8% of patients. In the last few years, updated guidelines on the management of bronchiolitis have been published, but none focused on critically ill infants. The American Academy of Pediatrics guidelines recommend only supportive therapy, such as oxygen therapy for hypoxemia, respiratory support, and maintenance of hydration.^25^ Continuous positive air pressure (CPAP) has traditionally been used as the first‐line respiratory support in severe VRIs.^26^ Our hospital has a long and wide experience in the use of Helmet CPAP, that is now available both in emergency and pediatric wards. Early CPAP has been associated with a lower risk of intubation, a faster normalization of heart and respiratory rates, and an increase in the PaO_2_/FiO_2_ ratio already after the first hour of treatment.^27^

More recently, the administration of heated and humidified oxygen with HFNC has been shown to play an important role in reducing respiratory work, improving gas exchanges and avoiding endotracheal intubation.^28^ In terms of pharmacological strategies, there is limited scientific evidence on the use of specific antiviral therapies in children with severe respiratory infections. Oseltamivir is the only drug approved by the Food and Drug Administration (FDA) and the European Medicines Agency (EMA) for the treatment of influenza in pediatrics. In several studies, this neuraminidase inhibitor reduced the viral replication and the duration of symptoms if started in the first 24–72 h. Ribavirin is a nucleoside inhibitor approved by the FDA for the treatment of RSV. The authors of the American Academy of Pediatrics guideline for the management of RSV bronchiolitis in children suggest considering this drug only in selected situations, such as patients with severe disease or those who are at risk for severe disease (e.g., immunocompromised and/or hemodynamically significant cardiopulmonary disease).^29^ The efficacy of a combined therapy with ribavirin plus IVIG is controversial. RSV infections in adult hematopoietic stem‐cell transplantation recipients have been treated with ribavirin alone or in combination with IVIG, showing variable success.^30^ Based on these findings, 27 patients of our cohort received oral ribavirin, combined in 19 patients with IVIG. Although no conclusions can be drawn in terms of efficacy because of a case–control study was not performed, we can state that none of these patients died or had life‐threatening side effects. One patient experienced an important increase of creatine phosphokinase blood level; however, this adverse event has never been described before during the treatment with ribavirin. A single‐case report of an adult patient presenting with rhabdomyolysis after the association with daptomycin, pegylated interferon α‐2b, and ribavirin was described by Colomba *et al*, but the author concluded that ribavirin did not play any role in the pathogenesis of the myopathy in that patient.^31^ Therefore, even though it is difficult to assess the cause of rhabdomyolysis in our patient, it appears unlikely to be related to the treatment with ribavirin.

Severe bronchiolitis requiring admission to PICU is frequently associated with morbidity; however, the mortality rate is low. In 2005, Panickar *et al* reported that mortality rates have fallen in the last three decades from 21.5 to 1.8 per 100.000 children below 12 months, reflecting improvements in pediatric intensive care.^32^ In a more recent review, death due to respiratory failure in bronchiolitis ranges from 2.9 (United Kingdom) to 5.3 (United States) deaths per 100.000 children.^33^ In our cohort, similar to data reported by Ghazaly and Nadel, death occurred in a very low rate of patients (1%).^13^

In conclusion, demographic and clinical variables can help to identify children affected by VRI at risk for worse outcome. Further studies are needed to design targeted interventions on high‐risk patient groups to reduce the risk of complications related to orotracheal intubation and prolonged hospitalization in intensive care settings.

## CONFLICT OF INTEREST

The authors declare that they have no competing interests.

## AUTHOR CONTRIBUTIONS

**Maia De Luca:** Conceptualization; data curation; methodology; project administration. **Carmen D'Amore:** Conceptualization; formal analysis; methodology. **Lorenza Romani:** Conceptualization; investigation. **Costanza Tripiciano:** Data curation. **Vitangelo Clemente:** Data curation. **Stefania Mercadante:** Data curation. **Daniela Perrotta:** Conceptualization; investigation; supervision. **Joseph Nunziata:** Conceptualization; investigation; supervision. **Corrado Cecchetti:** Conceptualization; investigation; supervision. **Emanuele Rossetti:** Conceptualization; investigation; supervision. **Roberto Bianchi:** Conceptualization; investigation; supervision. **Carlo Federico Perno:** Conceptualization; data curation; supervision. **Paola Bernaschi:** Conceptualization; data curation; supervision. **Cristina Russo:** Conceptualization; data curation; supervision. **Massimiliano Raponi:** Conceptualization; supervision. **Laura Lancella:** Conceptualization; supervision. **Marta Luisa Ciofi degli Atti:** Conceptualization; supervision.

## Data Availability

All data generated or analyzed during this study are included in this published article.
